# Eating Attitudes and Characteristics of Physical Activity Practitioners and Athletes in Riyadh, Saudi Arabia

**DOI:** 10.3390/healthcare12232439

**Published:** 2024-12-04

**Authors:** Reem S. Albassam, Alaa K. Alahmadi, Waad A. Alfawaz

**Affiliations:** Department of Community Health Sciences, College of Applied Medical Sciences, King Saud University, P.O. Box 10219, Riyadh 11433, Saudi Arabia; alaakhalidalahmadi@gmail.com (A.K.A.); walfawaz@ksu.edu.sa (W.A.A.)

**Keywords:** physical activity practitioners, athletes, eating attitude, disordered eating, nutritional knowledge, sports nutrition

## Abstract

**Background:** Disordered eating among athletes and physical activity practitioners is a growing concern that can negatively impact health and performance. Understanding the prevalence and predictors of disordered eating in these populations is essential for developing effective interventions. **Objective:** To investigate disordered eating tendencies among athletes and physical activity practitioners in Riyadh, Saudi Arabia. **Methods:** Participants from five athletic clubs and six fitness centers were surveyed. Data were collected using an interviewer-administered questionnaire that included the Eating Attitudes Test (EAT-26), the Abridged Nutrition for Sport Knowledge Questionnaire (ANSKQ), and the Global Physical Activity Questionnaire (GPAQ). Anthropometric data were also collected. The study comprised a sample of 263 individuals, who were divided into two groups: athletes (n = 121) and physical activity practitioners (n = 142). **Results:** The average age of the participants was 26.41 years with a standard deviation of 8.1 years. Females comprised nearly two thirds of the sample, representing 60.8% of the participants. Physical activity practitioners exhibited higher disordered eating scores compared to athletes. The total EAT-26 scores were significantly higher in practitioners (15.63 ± 4.12) than in athletes (13.21 ± 3.89; *p* < 0.001). Subscale scores for Dieting (8.95 ± 2.73 vs. 7.49 ± 2.58; *p* = 0.001) and Bulimia (3.32 ± 1.15 vs. 2.20 ± 1.03; *p* < 0.001) were also higher in practitioners. BMI was a significant predictor of higher total EAT-26 scores and its subsets, indicating a greater risk of disordered eating behaviors in individuals with higher BMI. Participants with lower levels of sports nutritional knowledge and those engaging in low-intensity physical activities were more likely to exhibit disordered eating tendencies. **Conclusions:** The findings highlight that physical activity practitioners are at a higher risk of disordered eating compared to athletes. Addressing BMI and improving nutritional knowledge are key strategies for preventing and managing disordered eating behaviors in physically active populations. Interventions targeting these areas may enhance health outcomes and performance among athletes and practitioners.

## 1. Introduction

Eating attitude problems are often described as a continuum ranging from disordered eating to a clinical eating disorder [1,2]. Disordered eating includes various symptoms of unhealthy eating patterns, such as fasting, restrictive dieting, self-induced vomiting, overeating, binge eating, and the misuse of laxatives or diet pills [3]. Disordered eating can lead to eating disorders if left unaddressed. The three main diagnostic categories of eating disorders are bulimia nervosa (BN), anorexia nervosa (AN), and binge eating disorder (BED). Other eating disorders, referred to as “atypical” forms, are classified under Specified Feeding and Eating Disorders (OSFEDs), which are feeding and eating disorders that cause clinically significant distress or impairment in social functioning but do not meet the full criteria for the three typical eating disorders (including atypical AN, BN, and BED; purging disorder; and night eating syndrome). [2,4] These disorders can lead to numerous negative mental and physical health effects [4,5].

Between 2000 and 2018, the global prevalence of eating disorders increased from 3.4% to 7.8% [4]. In western Asia, disordered eating behaviors are widespread with overall prevalence rates slightly higher than the international averages. According to assessments using various instruments, the reported prevalence rates are EAT-26/40 (22.07%), SCOFF (22.8%), EDDS (18.91%), and EDE-Q (7.95%) [6]. Estimates of the prevalence of disordered eating in Saudi Arabia vary widely in research due to the different populations studied (ages, gender, body weight, dietary intake, etc.). A recent systematic review of Saudi adolescents and young adults indicated a high prevalence of eating disorders and disordered eating behaviors, ranging from 10.2% to 48.1% [7].

Eating disorders arise from a complex combination of factors, including genetic predispositions, psychological issues such as low self-esteem, anxiety, and perfectionism, sociocultural influences like societal pressures for thinness and media portrayals of dieting and weight loss, as well as other elements such as trauma or stressful life events and family dynamics that emphasize weight and appearance. These combined factors contribute to the development of disordered eating behaviors, highlighting the multifaceted nature of their underlying causes [1,5,8].

Saudi Arabia’s Vision 2030 emphasizes physical activity as a cornerstone for enhancing quality of life and overall health [9]. While moderate to high levels of physical activity are generally encouraged for promoting overall health and well-being, different sports activities can be associated with varying levels of eating disorder psychopathology [10]. These activities have been identified as both risk and protective factors for disordered eating and body image concerns [11]. There is growing concern about potential disordered eating among physical activity practitioners—those engaging in exercise for fitness, health, and physique improvement [12], and athletes—individuals exercising to enhance performance and participate in organized sports [13], which can undermine health and performance. Factors like body image, competitive pressures, and nutritional knowledge may contribute to an increase in these tendencies in these populations [10]. A previous study in the Eastern Province of Saudi Arabia on physical activity practitioners found a prevalence of eating disorder symptoms as high as 36.6% [14]. In the international context, some studies have reported a higher prevalence of disordered eating in athletes [15], while other research has reported no differences in disordered eating between athletes and physical activity practitioners [16,17]. Athletes often experience significant external pressures from coaches, teammates, and the demands of competition, which can contribute to the development of disordered eating [16]. Along the same lines, individuals who engage in physical activity for health or aesthetic reasons (PA practitioners) may experience self-imposed pressures and societal influences that can lead to disordered eating behaviors [5,18].

Given these findings, evaluations are necessary to effectively screen high-risk populations for disordered eating, considering various physical activity patterns. The current literature lacks an understanding of these dynamics within the Saudi Arabian context, considering the country’s developmental goals. This study is the first to evaluate disordered eating tendencies among athletes and compare them with physical activity practitioners in Riyadh, Saudi Arabia. The aim of this research is to investigate these relationships and identify predictive factors for eating disorders using the EAT-26 tool [19]. By addressing these knowledge gaps, the study seeks to contribute valuable insights for developing effective preventive and screening strategies as well as interventions that align with national health objectives.

## 2. Materials and Methods

This study was reported in accordance with the Strengthening the Reporting of Observational Studies in Epidemiology (STROBE) guidelines [20].

### 2.1. Study Design and Population

This descriptive cross-sectional study focuses on athletes and physical activity practitioners at fitness centers and athletic clubs in Riyadh, Saudi Arabia. Athletes are defined as individuals exercising to enhance performance and participate in organized sports [13], while physical activity practitioners are those engaging in exercise for fitness, health, and physique improvement [12]. This study was conducted from June to December 2022, using convenient sampling. Participants were recruited from five athletic clubs and six fitness centers, where they received an overview of the study and were assured that their personal information would be kept anonymous to ensure privacy and confidentiality. The inclusion criteria required individuals to be over 18 years old, willing to participate, and to have signed consent forms. After obtaining consent, the questionnaires were administered through face-to-face interviews (see Supplementary Data; Figure S1). To determine a statistically significant association between EAT-26 scores and characteristics of physical activity practitioners and athletes, a sample size of 264 subjects was calculated based on an expected odds ratio of 2.0 with 80% power at a 0.05 significance level [21].

### 2.2. Data Collection

Data were collected via a face-to-face interview using a questionnaire divided into five sections:

- **Demographics**: Information on age, gender, social status, education level, income level, exercise motivation, intensity of exercise (classified by intensity levels), duration of exercise, and sources of nutritional information.
- **Anthropometry**: Weight (kg) and height (cm) were recorded, and body mass index (BMI) was calculated.
- **Nutritional Knowledge**: The Arabic Abridged Nutrition for Sport Knowledge Questionnaire (Arabic ANSKQ) assessed nutritional knowledge with response options including agree/disagree/not sure, high/low/not sure, and enough/not enough/not sure [22]. Scores were converted to percentages and categorized as follows: poor (0–49%), average (50–65%), good (66–75%), and excellent (above 75%) [22].
- **Physical Activity Levels**: The Arabic version of the Global Physical Activity Questionnaire (GPAQ) evaluated physical activity, categorizing scores into low, moderate, or high based on specific scoring criteria regarding the frequency and duration of physical activities [23].
- **Eating Attitudes**: The Eating Attitudes Test (EAT-26) is a widely used 26-item tool designed to evaluate attitudes and behaviors associated with disordered eating. It is divided into two main sections:
  1. Assessment of Potential Eating Disorders: This section uses a six-point Likert scale ranging from 0 to 3. To calculate the EAT-26 score, responses to items 1–25 are re-scored on a 4-point [Always (3), Usually (2), Often (1), Sometimes (0), Rarely (0), and Never (0)]. Item 24 is reverse scored. The total score is obtained by summing up the scores for all 26 items with a score of 20 or higher indicating a significant concern regarding disordered eating behaviors. This section includes three subscales that focus on specific aspects of eating behavior. The *Dieting Subscale* measures the deliberate avoidance of high-calorie foods, a focus on weight loss, and concerns about body image, shape, and size. It includes 13 questions with scores ranging from 0 to 39. The *Bulimia and Food Preoccupation Subscale* captures food-related thoughts and behaviors, such as binge eating and induced vomiting. It includes 6 questions, with scores ranging from 0 to 18. The *Oral Control Subscale* assesses self-regulation of eating habits and perceived external pressures related to weight gain. It includes 7 questions with scores ranging from 0 to 21.
  2. Eating Disorder Practices: This section consists of four behavioral questions assessing the frequency of disordered eating behaviors over the past six months. Participants who answer “yes” to any of these questions are encouraged to seek professional evaluation [19]. The EAT-26 demonstrates high reliability, with a Cronbach’s alpha of 0.908, making it a robust tool for identifying eating attitudes and potential disordered eating behaviors [24].

### 2.3. Statistical Analysis

The Kolmogorov–Smirnov and Shapiro–Wilk tests were used to assess the normality of quantitative outcome variables (EAT-26 scores). Since the data were normally distributed, parametric tests were applied in the analysis. For categorical study variables, Pearson’s Chi-square test was used to examine the effects of participant characteristics on their responses to the EAT-26 Potential Eating Disorders and Eating Disorder Practices Scale. In the bivariate analysis, Student’s *t*-test for independent samples and ANOVA were used to compare participant characteristics with the scores on the three subscales of the EAT-26 (Dieting, Bulimia, and Oral Control) as well as the total EAT-26 score. When ANOVA results were significant, a post hoc Tukey–Kramer test was employed to identify which conditions differed significantly from each other. Additionally, multiple regression analysis was conducted to examine the correlation between EAT-26 scores and characteristics of physical activity practitioners and athletes. A *p*-value of <0.05 was used to determine statistical significance. Data were analyzed using the Statistical Package for the Social Sciences (SPSS) version 28.0 software (IBM Inc., Armonk, NY, USA).

## 3. Results

A total of 263 participants were included in the study, consisting of 121 athletes and 142 physical activity practitioners. The average age of the subjects was 26.41 years with a standard deviation of 8.1 years. Nearly two thirds of the sample were female (60.8%), and a high proportion were single (73.4%). The characteristics of the study participants and the relationship between these characteristics and their response levels on the potential eating disorders scale and eating disorder practices according to the EAT-26 scale are presented in Table 1. The results revealed statistically significant differences in the responses of sports participants regarding potential eating disorders with 10.74% of athletes and 30.28% of casual sports practitioners displaying signs of potential eating disorders (*p*-value < 0.001).

There were also differences in body mass index classifications, with 55.13% of participants being overweight or obese, while only 7.65% had a normal weight (*p*-value < 0.001). Additionally, 57.69% of participants classified as overweight or obese, compared to 40% of those with normal weight, were reported engaging in eating disorder practices (*p*-value = 0.029 < 0.05).

Furthermore, there were statistically significant differences in sports nutritional knowledge regarding potential eating disorders. Of those with low nutritional knowledge, 24.75% were at risk for potential eating disorders compared to 9.26% of those with an acceptable level of knowledge (*p*-value = 0.043 < 0.05).

The results did not show any statistically significant differences (*p*-value > 0.05) in potential eating disorders or eating disorder practices based on other participant characteristics such as gender, age groups, educational level, social status, exercise intensity, nutritional knowledge, or the number of years of exercise (Table 1).

The descriptive characteristics of respondents based on their scores for Dieting, Bulimia, Oral Control, and the Total EAT-26 score are presented in Table 2. Each category provides insights into different eating attitudes and behaviors. Where regarding potential eating disorders, respondents without potential eating disorders had lower mean scores across the Dieting, Bulimia, and Total EAT-26 sections, except for the Oral Control section, compared to those with potential eating disorders (*p*-value < 0.05). Practitioners had significantly higher scores for Dieting (8.95 vs. 7.49, *p* = 0.001) and Bulimia (3.32 vs. 2.20, *p* < 0.001) compared to athletes. However, there were no significant differences in Oral Control (3.35 vs. 3.53, *p*-value = 0.256). Practitioners also had a higher Total EAT-26 score (15.63 vs. 13.21, *p* < 0.001). Although the differences in Dieting, Bulimia, and Oral Control scores were not statistically significant, males had a significantly higher Total EAT-26 score (15.48 vs. 13.90, *p* < 0.05).

Furthermore, younger respondents (18–25) had significantly lower Dieting scores (7.68, *p* = 0.006) and higher Oral Control scores (3.80, *p* = 0.007) compared to older age groups. Married participants had significantly higher scores for Dieting (9.67 vs. 7.80, *p* < 0.001), Bulimia (3.31 vs. 2.63, *p* = 0.026 < 0.05), and Total EAT-26 score (16.07 vs. 13.98, *p* < 0.05). There were no significant differences in Dieting, Bulimia, Oral Control, or Total EAT-26 scores based on education level.

Additionally, there were highly significant differences in scores across BMI categories, with overweight and obese individuals scoring much higher on Dieting (12.40), Bulimia (4.96), and Total EAT-26 (19.74), compared to normal weight and underweight individuals (*p* < 0.001 for all categories). There were no significant differences in Dieting, Bulimia, Oral Control, or Total EAT-26 scores based on the number of years participants had been playing sports. Moreover, those engaging in low-intensity sports had higher Dieting scores (9.31, *p* < 0.05) compared to those in medium and high-intensity sports. However, there were no significant differences in other categories.

Regarding nutritional knowledge, there were no significant differences in Dieting, Bulimia, or Total EAT-26 scores based on general nutritional knowledge (Table 2).

The predictive factors for the EAT-26 scores and its subsections (Dieting, Bulimia, Oral Control) based on participant characteristics such as BMI, reasons for exercise, age, type of sports played, sports nutritional knowledge, and weight change are presented in Table 3.

EAT-26 Total Score: In Model 1, the explanatory power for the total EAT-26 score is 25.5% (adjusted R^2^ = 0.252). BMI is a strong significant predictor, with a positive association (ß = 0.646, *p* < 0.001), indicating that higher BMI is linked to higher EAT-26 scores. In Model 2, the inclusion of additional variables (age, reason for exercise, type of sport, etc.) slightly improves the model’s explanatory power (R^2^ = 0.302, adjusted R^2^ = 0.282). While BMI remains a strong predictor (ß = 0.582, *p* < 0.001), sports nutritional knowledge emerges as a significant negative predictor (ß = −0.061, *p* = 0.005), suggesting that better sports nutritional knowledge is associated with lower EAT-26 scores. Other factors, such as reason for exercise and type of sport, did not significantly contribute to Model 2.

EAT-26 Dieting: In Model 1, the explanatory power for the EAT-26 Dieting score is 40.6% (adjusted R^2^ = 0.403). BMI is a strong positive predictor (ß = 0.549, *p* < 0.001), suggesting that higher BMI is associated with more dieting behaviors. In Model 2, the inclusion of additional variables improves the model’s explanatory power (R^2^ = 0.440, adjusted R^2^ = 0.424). In addition to BMI (ß = 0.465, *p* < 0.001), sports nutritional knowledge has a small but significant negative effect (ß = −0.028, *p* < 0.001), indicating that better nutritional knowledge reduces dieting behaviors. Weight change also appears as a significant predictor (ß = 0.055, *p* < 0.001), meaning those who experience weight changes are more likely to engage in dieting behaviors.

EAT-26 Bulimia: In Model 1, the model explains 30.1% of the variance in the EAT-26 Bulimia score (adjusted R^2^ = 0.298). BMI is a significant positive predictor (ß = 0.288, *p* < 0.001), indicating that higher BMI is associated with more bulimic behaviors. In Model 2, the model’s explanatory power increases slightly (R^2^ = 0.341, adjusted R^2^ = 0.323). BMI remains a significant predictor (ß = 0.297, *p* < 0.001), and the reason for exercise also becomes significant (ß = −0.512, *p* = 0.036), showing that athletes are less likely to exhibit bulimic behaviors compared to practitioners. Age and weight change do not significantly predict bulimia behaviors.

EAT-26 Oral Control: In Model 1, the model explains 13.3% of the variance in the EAT-26 Oral Control score (adjusted R^2^ = 0.130). BMI is negatively associated with Oral Control (ß = −0.192, *p* < 0.001), indicating that higher BMI is linked to lower Oral Control. In Model 2, the model’s explanatory power slightly increases (R^2^ = 0.154, adjusted R^2^ = 0.131). BMI remains a significant negative predictor (ß = −0.179, *p* < 0.001), and no other factors, including reason for exercise, age, type of sports played, or nutritional knowledge, have a significant impact on Oral Control (Table 3).

## 4. Discussion

This study aimed to assess and compare disordered eating tendencies among physical activity practitioners and athletes in Riyadh, Saudi Arabia. The findings of this study provide important insights into the prevalence and factors associated with disordered eating behaviors among different groups, particularly when using the Eating Attitudes Test (EAT-26) as a measure.

### 4.1. Socio-Demographic Characteristics and Disordered Eating Behaviors

Disorder eating behaviors measured by EAT-26 revealed that athletes (10.74%, 13.21 ± 4.8) and physical activity practitioners (30.28%, 15.63 ± 5.4) displayed signs of potential eating disorders with statistically significant differences (*p*-value < 0.001). This is supported by multiple studies where non-athletes showed more disordered eating behaviors than athletes, particularly gymnasts [16,17]. The higher prevalence of disordered eating behaviors among non-athletes compared to athletes can be attributed to several factors. Non-athletes often face greater societal pressures to conform to unrealistic beauty standards, leading to body dissatisfaction [16]. Additionally, they may lack the structured support systems and resources available to athletes. Furthermore, non-athletes are more likely to use disordered eating as a maladaptive coping mechanism to deal with stress, anxiety, or negative emotions. In contrast, athletes benefit from a structured environment, support systems [5], and the stress-reducing effects of exercise, which may help protect them from developing disordered eating behaviors.

### 4.2. BMI and Disordered Eating Behaviors

Across all models examined, BMI emerged as a significant and consistent predictor of disordered eating behaviors. Higher BMI was strongly associated with higher scores on the EAT-26, indicating greater engagement in dieting (ß = 0.549, *p* < 0.001) and bulimic behaviors (ß = 0.288, *p* < 0.001). This finding aligns with previous studies that link higher BMI with an increased risk of disordered eating [25,26], which is often driven by societal pressures to conform to certain body ideals or as a response to the stigmatization of higher body weight [27]. A previous cross-sectional study revealed that females with obesity had the highest prevalence of eating disorders, while normal-weight females had the lowest prevalence [28]. Interestingly, while a higher BMI predicted more dieting and bulimic behaviors, it was associated with lower oral control, suggesting a complex relationship between body weight and eating behaviors where those with higher BMI may struggle more with controlling their eating impulses. While bulimia nervosa, which involves purging, often occurs in individuals with a normal BMI [2,4], the Bulimia subscale of the EAT-26 may capture food preoccupation and binge eating behaviors prevalent in those with higher BMI. Therefore, the observed association may be indicative of binge eating disorder behaviors rather than bulimia nervosa. Higher BMI was a significant predictor of disordered eating behaviors in males [29], which was similar to the findings in our study.

We found that weight change is a significant predictor in the dieting model (ß = 0.055, *p* < 0.001), suggesting that individuals who experience fluctuations in weight are more prone to engage in dieting behaviors. Research indicates a bidirectional relationship between weight fluctuations and dieting practices, potentially leading to adverse health outcomes. Weight cycling was observed to be prevalent among individuals engaged in physical activity, especially among females and those who self-reported as overweight [30]. Longitudinal studies highlight the link between changes in weight perception and dietary habits [31]. These findings emphasize the importance of considering BMI classification when screening for disordered eating, especially in individuals with higher BMI.

### 4.3. Impact of Sports Nutritional Knowledge on Disordered Eating Behaviors

The analysis also highlights the protective role of sports nutritional knowledge in mitigating disordered eating behaviors, particularly in the context of dieting (ß = −0.028, *p* < 0.001). Participants with better sports-specific nutritional knowledge were less likely to engage in extreme dieting behaviors, as reflected in lower EAT-26 scores. This suggests that education and awareness around nutrition, especially within the context of sports, can play a critical role in promoting healthier eating habits and reducing the risk of disordered eating. A recent study was conducted to understand the level of nutritional knowledge among the athletic population in Riyadh, Saudi Arabia, and the researchers found that most participants had poor nutritional knowledge; those findings contribute to the growing evidence of the essential role of nutritional knowledge in influencing food choices and eating habits [32]. Previous research has indicated a correlation between inadequate nutritional knowledge and an increased risk of developing components of the female athlete triad, including disordered eating behaviors [33]. Insufficient sports nutrition knowledge, particularly among female athletes, is associated with disordered eating behaviors and poor dietary adherence [34,35]. Our study reinforces this connection, showing that lower sports nutrition knowledge correlates with higher disordered eating scores. This highlights the potential of educational interventions to mitigate the risk of disordered eating in this population.

Educational interventions have demonstrated their effectiveness in reducing eating disorder risk factors among high-risk groups [36]. This aligns with our findings on the protective role of sports nutritional knowledge, suggesting that targeted educational programs can be effective in reducing disordered eating behaviors. The success of such interventions supports the idea that increasing awareness and knowledge about nutrition, particularly in sports and exercise contexts, could be a key strategy in prevention efforts.

### 4.4. Impact of Exercise Type and Intensity on Disordered Eating Behaviors

The type of exercise and the reasons for engaging in it also appeared to influence eating behaviors, though to a lesser extent than BMI and nutritional knowledge. Specifically, athletes were found to have lower bulimic behaviors compared to physical activity practitioners, suggesting that structured and competitive sports environments may offer some protective benefits against these behaviors. This could be attributed to the focus on overall health and performance, rather than solely physical appearance, in more competitive settings. However, it is important to note that this protective effect did not extend to other aspects of disordered eating, such as dieting or oral control, indicating that the relationship between exercise and eating behaviors is multifaceted.

Adolescent female athletes, especially those involved in competitive, lean, non-aesthetic sports, are at increased risk of disordered eating, which is influenced by factors like competition outcomes, training intensity, and individual motivations for exercise [15,37,38].

Interestingly, other demographic factors such as age, gender, and educational level showed limited or no significant impact on the prevalence of disordered eating behaviors. This suggests that while these factors are often considered in the literature, their influence may be overshadowed by more immediate and modifiable factors like BMI and nutritional knowledge.

### 4.5. Gender Differences in Disordered Eating Behaviors

Both males and females can develop eating disorders; however, the nature of these disorders often differs by gender. In a previous cross-sectional study, the prevalence of extreme dieting and purging was higher in males compared to females [39]. Another study found that weight/appearance-related anxiety was a significant risk factor for both males and females [40]. Disordered eating behaviors are common among both males and females with some gender differences in specific behaviors [41]. Eating disorders and disordered eating behaviors in males may present differently than in females, particularly with muscularity-oriented disordered eating [42]. Our study found that males had a higher score on all subscales, but the significant gender difference was in total EAT-26 scores only.

### 4.6. Age in Disordered Eating Behaviors

Disordered eating behaviors can vary significantly across different life stages. Multiple studies have concluded that disordered eating behaviors decrease with age in females [43,44]. Longitudinal studies indicate a persistent and escalating trend of dieting and disordered eating behaviors from adolescence to young adulthood with individuals exhibiting consistent engagement in these practices over time [45]. Gender differences in eating disorder psychopathology diminish over time from late adolescence to later midlife [46]. Notably, eating disorder symptoms exhibit a marked increase during the ages of 12 to 15, underscoring the critical importance of early prevention initiatives [47]. While our study found significant age-related differences in eating behaviors especially dieting in older adults (9.61, *p* = 0.006) and oral control in younger groups (3.80, *p* = 0.007), this suggests that the manifestation and severity of disordered eating behaviors are not static and may evolve across the lifespan, necessitating tailored prevention and intervention strategies at different stages of development.

The findings of this study represent a crucial initial step in identifying risk factors for disordered eating behaviors within the Saudi Arabian athletic community, particularly as physical activity levels increase in alignment with the 2030 Vision. These insights will facilitate the development of targeted intervention programs and provide healthcare professionals with a preliminary understanding of high-risk individuals, enabling early prevention and intervention strategies for disordered eating behaviors.

### 4.7. Limitations

This study has several limitations. Its cross-sectional design provides a snapshot of the population at a specific time, preventing the establishment of causal relationships. The study’s scope is limited to Riyadh, Saudi Arabia, necessitating further research to generalize findings to other Arab populations. Future studies with larger, more diverse samples, including elite and esthetic athletes, are needed to confirm these findings. While BMI is a widely used anthropometric measure, it has limitations for individuals with high muscle mass or fluid retention. Additionally, BMI does not account for regional body fat distribution. Future research may benefit from using BMI in conjunction with waist circumference.

The study’s strengths include rigorous data collection methods. Comprehensive researcher training, face-to-face interviews (considered more accurate than self-administered questionnaires in diagnosing eating behaviors [16]), automated data entry, and the use of validated questionnaires enhance the credibility and significance of the findings. As the first study to evaluate disordered eating tendencies among athletes and compare them with physical activity practitioners in Riyadh, Saudi Arabia, it provides valuable insights into this population.

### 4.8. Future Implications

*Nutritional Education:* Our findings have linked sports nutritional knowledge to lower incidences of disorders eating behaviors, highlighting the importance of incorporating comprehensive nutritional education into training programs for athletes and physical activity practitioners. This aids in reducing the prevalence of eating disorders among these populations.

*Public Health Interventions:* Findings that being overweight or obese and weight cycling increase the risk for disordered eating behaviors emphasize the need for public health strategies that accentuate healthy weight management. Interventions should not only foster a balanced approach to diet and exercise but also consider mental health aspects, such as body image and self-esteem. Targeted interventions addressing body image, stress management, and nutrition education can be designed to mitigate the risk of disordered eating in athletic populations.

*Sports Community Practices:* The data show a higher tendency for eating disorders among recreational sports participants compared to competitive athletes. This suggests that recreational centers and gyms, as well as sports clubs and organizations, should consider routine psychological and nutritional screenings to identify individuals at risk and provide appropriate support or referrals.

*Healthcare setting:* Collaborative efforts among healthcare providers, sports medicine specialists, and mental health professionals are crucial to provide comprehensive care for individuals struggling with disordered eating. Routine screening for disordered eating should be integrated into health assessments for athletes and practitioners to enable early detection and intervention.

*Research:* Future research should investigate deeper into the psychological roots of disordered eating among physical activity practitioners, focusing on factors such as body image issues, perfectionism, stress, mood, depression, and motivation. Longitudinal studies would be valuable to understand whether these disorders tendencies persist or change over time and to assess the long-term health and performance consequences of disordered eating in athletes and practitioners.

## 5. Conclusions

The findings reinforce the importance of addressing BMI and improving nutritional knowledge as key strategies in the prevention and management of disordered eating behaviors, particularly in populations engaged in regular physical activity. The consistent link between BMI and disordered eating across different populations highlights the need for interventions, targeting weight management and body image perceptions, which could be instrumental in reducing the prevalence of eating disorders. Future research should continue to explore the complex interplay between these variables to better inform public health strategies and clinical practices.

## Figures and Tables

**Table 1:** Descriptive characteristics of respondents based on their responses to the EAT26.

	“Potential Eating Disorders” EAT 26			“Eating Disorder Practices”		
	EAT 26 < 20 (n = 207)	EAT 26 > 20 (n = 56)	*p*-Value	No (n = 142)	Yes (n = 121)	*p*-Value
**Reason for exercise**						
Practitioner	99 (69.72%)	43 (30.28%)	<0.001 ***	73 (51.41%)	69 (48.59%)	0.362
Athletes	108 (89.26%)	13 (10.74%)		69 (57.02%)	52 (42.98%)	
**Gender**						
Male	75 (72.82%)	28 (27.18%)	0.061	49 (47.57%)	54 (52.43%)	0.094
Female	132 (82.5%)	28 (17.5%)		93 (58.13%)	67 (41.88%)	
**Age (years)**						
18–25	113 (79.58%)	29 (20.42%)	0.740	78 (54.93%)	64 (45.07%)	0.924
26–35	67 (76.14%)	21 (23.86%)		46 (52.27%)	42 (47.73%)	
>35	27 (81.82%)	6 (18.18%)		18 (54.55%)	15 (45.45%)	
**Social Status**						
Single	158 (80.61%)	38 (19.39%)	0.197	110 (56.12%)	86 (43.88%)	0.236
Married	49 (73.13%)	18 (26.87%)		32 (47.76%)	35 (52.24%)	
**Education level**						
No higher education	68 (78.16%)	19 (21.84%)	0.879	44 (50.57%)	43 (49.43%)	0.434
Higher education	139 (78.98%)	37 (21.02%)		98 (55.68%)	78 (44.32%)	
**Type of sports played**						
Low intensity	31 (68.89%)	14 (31.11%)	0.060	25 (55.56%)	20 (44.44%)	0.750
Medium intensity	79 (85.87%)	13 (14.13%)		52 (56.52%)	40 (43.48%)	
High intensity	97 (76.98%)	29 (23.02%)		65 (51.59%)	61 (48.41%)	
**BMI**						
Underweight	15 (100%)	0 (0%)	<0.001 ***	7 (46.67%)	8 (53.33%)	0.029 *
Normal weight	157 (92.35%)	13 (7.65%)		102 (60%)	68 (40%)	
Overweight and Obese	35 (44.87%)	43 (55.13%)		33 (42.31%)	45 (57.69%)	
**Nutritional knowledge**						
Poor	154 (75.86%)	49 (24.14%)	0.115	107 (52.71%)	96 (47.29%)	0.225
Fair	47 (88.68%)	6 (11.32%)		29 (54.72%)	24 (45.28%)	
Good	6 (85.71%)	1 (14.29%)		6 (85.71%)	1 (14.29%)	
**Sports Nutritional knowledge**						
Poor	152 (75.25%)	50 (24.75%)	0.043 *	101 (50%)	101 (50%)	0.052
Fair	49 (90.74%)	5 (9.26%)		37 (68.52%)	17 (31.48%)	
Good	6 (85.71%)	1 (14.29%)		4 (57.14%)	3 (42.86%)	
**Years of playing sports**						
≤2	69 (76.67%)	21 (23.33%)	0.804	52 (57.78%)	38 (42.22%)	0.122
3–5	69 (82.14%)	15 (17.86%)		51 (60.71%)	33 (39.29%)	
6–9	25 (75.76%)	8 (24.24%)		14 (42.42%)	19 (57.58%)	
>9	44 (78.57%)	12 (21.43%)		25 (44.64%)	31 (55.36%)	

**Note: “Potential Eating Disorders”**: A score of 20 or higher on the EAT-26 indicates a high level of concern about dieting, body weight, or problematic eating behaviors. **“Eating Disorder Practices”**: If the participant answered “yes” to any behavioral question, they should seek an evaluation from a trained mental health professional specializing in the treatment of eating disorders. *** *p* value < 0.001, * *p* value < 0.05.

**Table 2:** Descriptive characteristics of respondents based on their scores for Dieting, Bulimia, Oral Control, and Total EAT-26 score.

	EAT-26 Dieting		EAT-26 Bulimia		EAT-26 Oral Control		EAT-26 Total	
	Mean (SD)	*p*-Value	Mean (SD)	*p*-Value	Mean (SD)	*p*-Value	Mean (SD)	*p*-Value
**Eating Attitudes**								
Without potential eating disorders	7.08 (2.86)	0.001 **	1.99 (1.4)	<0.001 ***	3.36 (2.2)	0.276	12.45 (3.65)	<0.001 ***
With potential eating disorders	12.7 (2.02)		5.84 (1.76)		3.71 (2.04)		22.25 (2.22)	
**Reason for exercise**								
Practitioner	8.95 (3.8)	0.001 *	3.32 (2.3)	<0.001 ***	3.35 (2)	0.256	15.63 (5.4)	<0.001 ***
Athletes	7.49 (3)		2.2 (1.9)		3.53 (2.3)		13.21 (4.8)	
**Gender**								
Male	8.73 (3.4)	0.099	3.11 (2.4)	0.071	3.64 (2.2)	0.107	15.48 (5.5)	0.018 *
Female	7.99 (3.6)		2.61 (2)		3.3 (2.2)		13.9 (5.1)	
**Age (years)**								
18–25	7.68 (3.4)	0.006 **	2.71 (2.1)	0.405	3.8 (2.3)	0.007 **	14.2 (5)	0.388
26–35	8.74 (3.6)		2.78 (2.2)		3.11 (2.2)		14.64 (5.7)	
>35	9.61 (3.5)		3.27 (2.3)		2.7 (1.4)		15.58 (5.2)	
**Social Status**								
Unmarried	7.8 (3.5)	<0.001 ***	2.63 (2.1)	0.026 *	3.55 (2.2)	0.067	13.98 (5.2)	0.005 **
Married	9.67 (3.5)		3.31 (2.3)		3.09 (2)		16.07 (5.3)	
**Education level**								
No higher education	8.01 (3.5)	0.394	2.83 (2.2)	0.910	3.53 (2.2)	0.309	14.37 (4.9)	0.747
Higher education	8.41 (3.6)		2.8 (2.1)		3.39 (2.2)		14.59 (5.4)	
**BMI**								
Underweight	3.53 (0.8)	<0.001 ***	1.47 (1.1)	<0.001 ***	7.33 (1.3)	<0.001 ***	12.33 (2.3)	<0.001 ***
Normal weight	6.81 (2.3)		1.94 (1.4)		3.57 (2)		12.31 (4.3)	
Overweight and obese	12.4 (2.1)		4.96 (2.2)		2.38 (1.8)		19.74 (3.6)	
**Years of playing sports**								
≤2	8.34 (3.7)	0.343	2.97 (2.3)	0.304	3.53 (1.9)	0.523	14.84 (5.3)	0.286
3–5	7.75 (3.7)		2.44 (1.9)		3.46 (2.5)		13.65 (5.2)	
6–9	8.52 (3)		3.09 (2.4)		2.91 (2)		14.52 (5.4)	
>9	8.82 (3.3)		2.93 (2.1)		3.54 (2.1)		15.29 (5.1)	
**Type of sports played**								
Low intensity	9.31 (4)	0.034 *	3.2 (2.1)	0.108	3.27 (2.3)	0.579	15.78 (5.8)	0.091
Medium intensity	7.65 (3)		2.45 (2.1)		3.62 (2.4)		13.72 (4.6)	
High intensity	8.37 (3.7)		2.93 (2.2)		3.36 (2)		14.65 (5.5)	
**Nutritional knowledge**								
Poor	8.34 (3.6)	0.860	2.89 (2.3)	0.485	3.57 (2.2)	0.145	14.8 (5.3)	0.282
Fair	8.04 (3.4)		2.49 (1.8)		3.06 (2.1)		13.58 (4.9)	
Good	8.29 (3.5)		2.71 (1.7)		2.43 (2)		13.43 (6.1)	
**Sports Nutritional knowledge**								
Poor	8.46 (3.6)	0.111	2.96 (2.3)	0.009 **	3.55 (2.2)	0.133	14.97 (5.3)	0.014 *
Fair	7.44 (3.3)		2.07 (1.6)		3.15 (1.9)		12.67 (4.8)	
Good	9.57 (3.5)		4 (2.1)		2.14 (2.2)		15.71 (5.8)	

Note: *** *p* value < 0.001, ** *p* value < 0.01, * *p* value < 0.05.

**Table 3:** Regression models for predicting EAT-26 scores and EAT-26 sections and characteristics of participants.

Items	ß	SE-b	Beta	t	*p*	95% CI		R^2^
						Lower	Upper	
**EAT-26 Total**								
Model 1								R^2^ = 0.255; adjusted R^2^ = 0.252.
(Constant)	−0.76	1.64		−0.463	0.644	−3.989	2.469	
BMI	0.646	0.068	0.505	9.455	**<0.001**	0.511	0.78	
Model 2								
(Constant)	3.842	2.075		1.852	0.065	−0.244	7.928	R^2^ = 0.302; adjusted R^2^ = 0.282.
BMI	0.582	0.083	0.456	7.054	**<0.001**	0.42	0.745	
Reason for exercise (Athletes) #	−0.663	0.608		−1.091	0.276	−1.861	0.534	
Age (years)	−0.042	0.037	−0.065	−1.136	0.257	−0.116	0.031	
Type of sports played (Medium intensity) ##	−0.407	0.863		−0.472	0.638	−2.107	1.293	
Type of sports played (High intensity) ##	0.017	0.831		0.021	0.984	−1.619	1.653	
Sports nutritional knowledge	−0.061	0.022	−0.156	−2.811	**0.005**	−0.103	−0.018	
Wt. change	0.052	0.03	0.108	1.7	0.09	−0.008	0.112	
**EAT** **-** **26 Dieting**								
Model 1								R^2^ = 0.406; adjusted R^2^ = 0.403.
(Constant)	−4.719	0.988		−4.775	**<0.001**	−6.665	−2.773	
BMI	0.549	0.041	0.637	13.349	**<0.001**	0.468	0.63	
Model 2								
(Constant)	−2.259	1.254		−1.801	0.073	−4.728	0.21	R^2^ = 0.44; adjusted R^2^ = 0.424.
BMI	0.465	0.05	0.539	9.313	**<0.001**	0.366	0.563	
Reason for exercise (Athletes) #	−0.013	0.367		−0.034	0.973	−0.736	0.711	
Age (years)	0.003	0.023	0.006	0.124	0.902	−0.042	0.047	
Type of sports played (Medium intensity) ##	−0.57	0.522		−1.093	0.275	−1.597	0.457	
Type of sports played (High intensity) ##	−0.201	0.502		−0.401	0.689	−1.19	0.788	
Sports nutritional knowledge	−0.028	0.013	−0.105	−2.115	**<0.001**	−0.053	−0.002	
Wt. change	0.055	0.018	0.17	2.996	**<0.001**	0.019	0.091	
**EAT-26 Bulimia**								
Model 1								R^2^ = 0.301; adjusted R^2^ = 0.298.
(Constant)	−4.018	0.653		−6.152	**<0.001**	−5.304	−2.732	
BMI	0.288	0.027	0.549	10.604	**<0.001**	0.235	0.342	
Model **2**								
(Intercept)	−2.767	0.829		−3.338	**<0.001**	−4.4	−1.135	R^2^ = 0.341; adjusted R^2^ = 0.323.
BMI	0.297	0.033	0.565	8.994	**<0.001**	0.232	0.362	
Reason for exercise (Athletes) #	−0.512	0.243		−2.109	**0.036**	−0.991	−0.034	
Age (years)	−0.025	0.015	−0.094	−1.681	0.094	−0.055	0.004	
Type of sports played (Medium intensity) ##	0.008	0.345		0.024	0.981	−0.671	0.687	
Type of sports played (High intensity) ##	0.209	0.332		0.63	0.529	−0.444	0.863	
Sports nutritional knowledge	−0.016	0.009	−0.098	−1.817	0.07	−0.033	0.001	
Wt. change	−0.004	0.012	−0.018	−0.299	0.765	−0.028	0.02	
**EAT-26 Oral Control**								
Model 1								R^2^ = 0.133; adjusted R^2^ = 0.13.
(Constant)	7.978	0.729		10.941	**<0.001**	6.542	9.414	
BMI	−0.192	0.03	−0.365	−6.326	**<0.001**	−0.252	−0.132	
Model **2**								R^2^ = 0.154; adjusted R^2^ = 0.131.
(Constant)	8.868	0.941		9.424	**<0.001**	7.015	10.721	
BMI	−0.179	0.037	−0.34	−4.78	**<0.001**	−0.253	−0.105	
Reason for exercise (Athletes) #	−0.139	0.276		−0.502	0.616	−0.682	0.405	
Age (years)	−0.02	0.017	−0.075	−1.188	0.236	−0.054	0.013	
Type of sports played (Medium intensity) ##	0.155	0.391		0.396	0.693	−0.616	0.926	
Type of sports played (High intensity) ##	0.009	0.377		0.024	0.981	−0.733	0.751	
Sports nutritional knowledge	−0.017	0.01	−0.109	−1.778	0.077	−0.037	0.002	
Wt. change	0.0002	0.014	0.001	0.02	0.984	−0.027	0.027	

# compared with Reason for exercise (Practitioner), ## compared with Type of sports played (Low intensity), ß = the standardized beta, SE-b = standard error unstandardized beta, B = unstandardized beta, *t* = *t*-test statistic, *p* = probability value.

## Data Availability

The datasets generated during and/or analyzed during the current study are not publicly available due to data protection requirements, but are available from the corresponding author upon reasonable request.

## Supplementary Materials

The following supporting information can be downloaded at https://www.mdpi.com/article/10.3390/healthcare12232439/s1, Figure S1. Data collection flowchart.
