# Supplementary figures and images for: Eating Attitudes and Characteristics of Physical Activity Practitioners and Athletes in Riyadh, Saudi Arabia
Source: Healthcare (Basel). 2024 Dec 4;12(23):2439. doi: 10.3390/healthcare12232439 (PMC11641735; doi:10.3390/healthcare12232439)

Figure S1. Data collection flowchart.

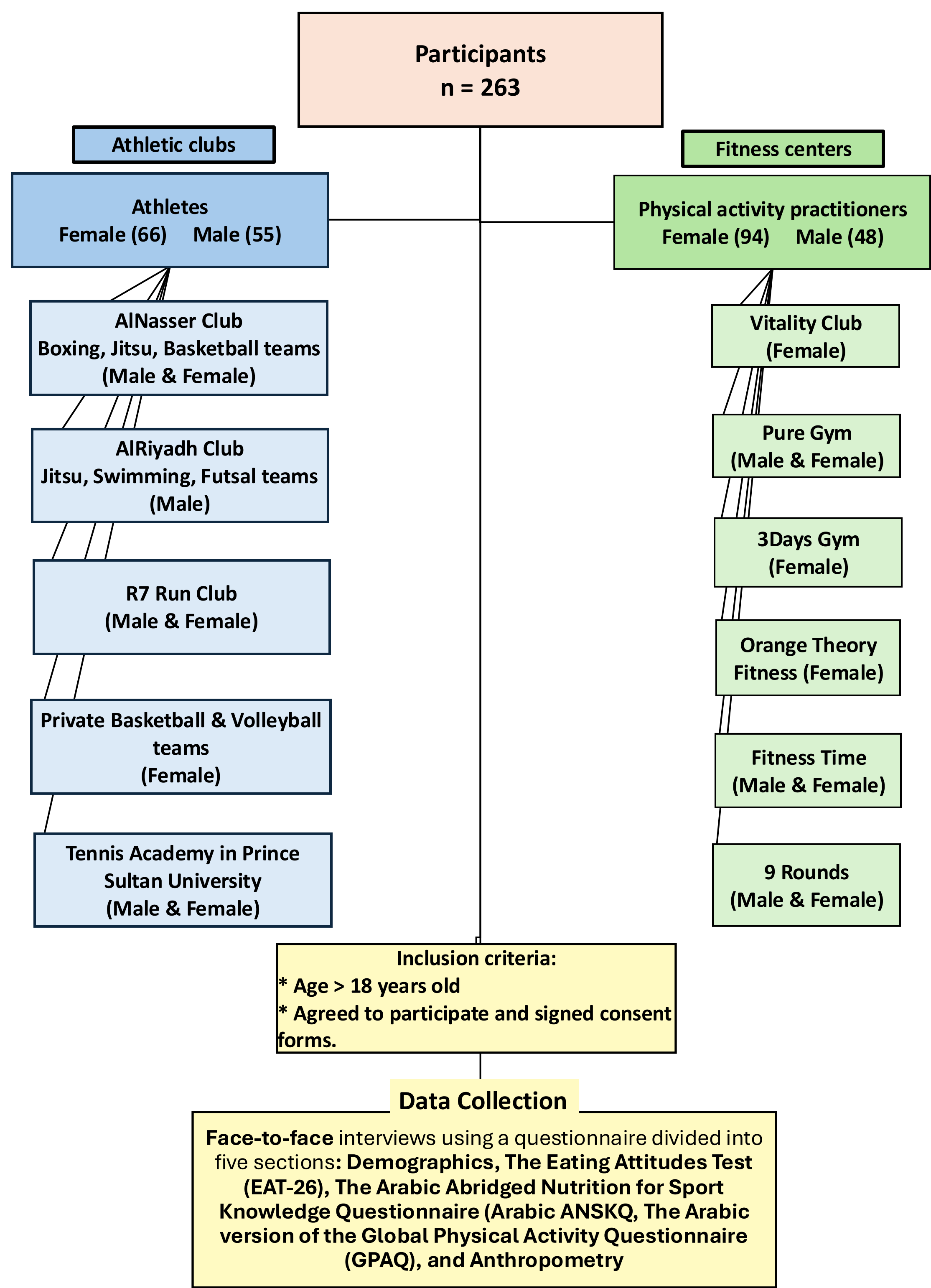

Supplement: Supplementary file 1 [file healthcare-12-02439-s001.zip › healthcare-3315723-supplementary.pdf]
